# Expression of Suppressor of Cytokine Signaling 1 (SOCS1) Impairs Viral Clearance and Exacerbates Lung Injury during Influenza Infection

**DOI:** 10.1371/journal.ppat.1004560

**Published:** 2014-12-11

**Authors:** Keer Sun, Sharon Salmon, Vijaya Kumar Yajjala, Christopher Bauer, Dennis W. Metzger

**Affiliations:** 1 Center for Immunology and Microbial Disease, Albany Medical College, Albany, New York, United States of America; 2 Department of Pathology and Microbiology, University of Nebraska Medical Center, Omaha, Nebraska, United States of America; National Institutes of Health, United States of America

## Abstract

Suppressor of cytokine signaling (SOCS) proteins are inducible feedback inhibitors of cytokine signaling. SOCS1^−/−^ mice die within three weeks postnatally due to IFN-γ-induced hyperinflammation. Since it is well established that IFN-γ is dispensable for protection against influenza infection, we generated SOCS1^−/−^IFN-γ^−/−^ mice to determine whether SOCS1 regulates antiviral immunity in vivo. Here we show that SOCS1^−/−^IFN-γ^−/−^ mice exhibited significantly enhanced resistance to influenza infection, as evidenced by improved viral clearance, attenuated acute lung damage, and consequently increased survival rates compared to either IFN-γ^−/−^ or WT animals. Enhanced viral clearance in SOCS1^−/−^IFN-γ^−/−^ mice coincided with a rapid onset of adaptive immune responses during acute infection, while their reduced lung injury was associated with decreased inflammatory cell infiltration at the resolution phase of infection. We further determined the contribution of SOCS1-deficient T cells to antiviral immunity. Anti-CD4 antibody treatment of SOCS1^−/−^IFN-γ^−/−^ mice had no significant effect on their enhanced resistance to influenza infection, while CD8^+^ splenocytes from SOCS1^−/−^IFN-γ^−/−^ mice were sufficient to rescue RAG1^−/−^ animals from an otherwise lethal infection. Surprisingly, despite their markedly reduced viral burdens, RAG1^−/−^ mice reconstituted with SOCS1^−/−^IFN-γ^−/−^ adaptive immune cells failed to ameliorate influenza-induced lung injury. In conclusion, in the absence of IFN-γ, the cytoplasmic protein SOCS1 not only inhibits adaptive antiviral immune responses but also exacerbates inflammatory lung damage. Importantly, these detrimental effects of SOCS1 are conveyed through discrete cell populations. Specifically, while SOCS1 expression in adaptive immune cells is sufficient to inhibit antiviral immunity, SOCS1 in innate/stromal cells is responsible for aggravated lung injury.

## Author Summary

Cytokines are critical in regulating the balance between protective immunity and detrimental inflammation during influenza infection. Suppressor of cytokine signaling (SOCS) proteins are inducible feedback inhibitors of cytokine signaling. Using gene-deficient and infectious animal models, we determined how SOCS1 regulates immune defense against influenza infection. We show that the intracellular protein SOCS1 not only inhibits adaptive antiviral immune responses but also exacerbates inflammatory lung damage. These detrimental effects of SOCS1 are conveyed through discrete cell populations. Specifically, while SOCS1 expression in adaptive immune cells is sufficient to inhibit antiviral immunity, SOCS1 in innate/stromal cells is responsible for aggravated lung injury. To our knowledge, there is no report showing the regulatory role of SOCS1 during the course of influenza infection, and importantly, no evidence directly linking SOCS1 with excessive inflammation in other infectious disease models. The distinct and non-competing detrimental roles of SOCS1, as revealed in this study, make it an appealing target in the design of effective immunotherapies for combating influenza infection.

## Introduction

Influenza virus causes highly contagious acute respiratory disease. Despite vaccine availability, the virus remains a major worldwide health problem. Proper host immunity is essential for virus clearance and recovery, with T cells playing a major role [1]. Cytokines have pivotal effects in the initiation and regulation of immune responses. In recent years, SOCS proteins have been identified as a negative feedback loop to attenuate cytokine signaling [2]–[4]. The induction of SOCS proteins by influenza virus has been recently reported; however, the role of these cytoplasmic proteins in immune defense against influenza infection remains unclear [5]–[7].

SOCS1 is a critical feedback inhibitor of both IFN-γ/STAT1 [8], [9] and IL-4/STAT6 signaling pathways [10], [11]. Due to its mutual suppression of both Th1 and Th2 responses, i.e., high IFN-γ levels inhibit IL-4/STAT6 signaling, whereas high levels of IL-4 suppress the IFN-γ/STAT1 pathway [12], IFN-γ-induced SOCS1 production could increase the threshold of T cell responsiveness to IL-4 [4], thereby facilitating the establishment of a Th1/IFN-γ-biased immune environment during influenza infection [13]. SOCS1^−/−^ mice die by postnatal week three due to IFN-γ-induced hyperinflammation [14], [15]. Although influenza infection induces strong T cell-dependent IFN-γ production, IFN-γ is dispensable for protective antiviral immunity [16], [17]. Therefore, we developed SOCS1^−/−^IFN-γ^−/−^ mice to evaluate the role of SOCS1 during influenza infection (S1 Figure). We found that SOCS1 deficiency not only enhanced viral clearance but also improved the resolution of acute inflammation. These findings were in stark contrast to observations in other infectious disease models where SOCS1-deficient mice, including SOCS1^−/−^IFN-γ^−/−^ and SOCS1^+/−^, demonstrated both enhanced IFN antimicrobial and detrimental pro-inflammatory activities [8], [18], [19]. Furthermore, here we demonstrate that these non-competing detrimental effects on host resistance to influenza infection are mediated by SOCS1 expression in different cell types. While SOCS1 in adaptive immune cells inhibits antiviral immunity, its presence in innate/stromal cells is responsible for aggravated lung damage.

## Results

### SOCS1 deficiency results in increased resistance to influenza infection

**Figure 1:**
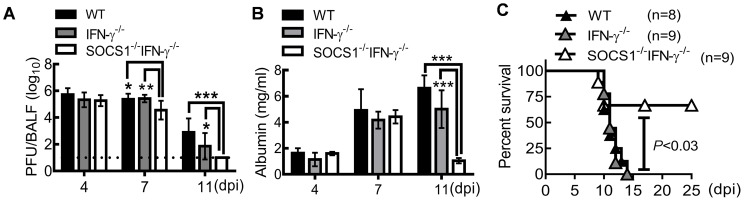
SOCS1^−/−^IFN-γ^−/−^ mice are more resistant to influenza infection. (**A**) Viral titers (6–9 mice/group), (**B**) albumin levels (4 mice/group), and (**C**) survival of C57BL/6 WT, IFN-γ^−/−^, and SOCS1^−/−^IFN-γ^−/−^ mice after i.n. infection of (**A, B**) 50 PFU or (**C**) 10^3^ PFU PR8 virus. In (A&B), *P*<0.001, ANOVA; *P<0.05, **, *P*<0.01, ***, *P*<0.001, Tukey's multiple comparisons test. Data in (A) were combined from two independent experiments. Data in (B&C) are representative of at least two experiments.

C57BL/6 WT, IFN-γ^−/−^ and SOCS1^−/−^IFN-γ^−/−^ mice were intranasally infected with A/PR/8 influenza virus to determine the regulatory effect of SOCS1 on host defense. Similar viral clearance kinetics were detected in WT and IFN-γ^−/−^ mice after a sublethal dose (50 PFU) of PR8 infection (Fig. 1A). This observation is consistent with other reports, which showed that IFN-γ is dispensable for immune defense against influenza infection [16], [17]. Although viral burdens at the early stage of infection (4 dpi) were comparable in WT and gene-deficient mice, SOCS1^−/−^IFN-γ^−/−^ mice exhibited improved viral clearance at 7 dpi compared to both IFN-γ^−/−^ and WT mice, as revealed by a10-fold decrease in viral burdens (Fig. 1A). We also assessed influenza-induced lung vascular injury by quantitating albumin efflux into the airway. In both WT and IFN-γ^−/−^ mice, albumin levels increased at 7 dpi and remained elevated at 11 dpi. In contrast, albumin concentrations in SOCS1^−/−^IFN-γ^−/−^ airways were significantly decreased at 11 dpi (Fig. 1B), indicating that SOCS1 deficiency is associated with attenuated lung vascular damage at the resolution phase of infection. Accordingly, SOCS1^−/−^IFN-γ^−/−^ mice exhibited improved survival rates after an otherwise lethal dose (1000 PFU) of PR8 infection (Fig. 1C).

### SOCS1 deficiency results in enhanced adaptive immune responses

**Figure 2:**
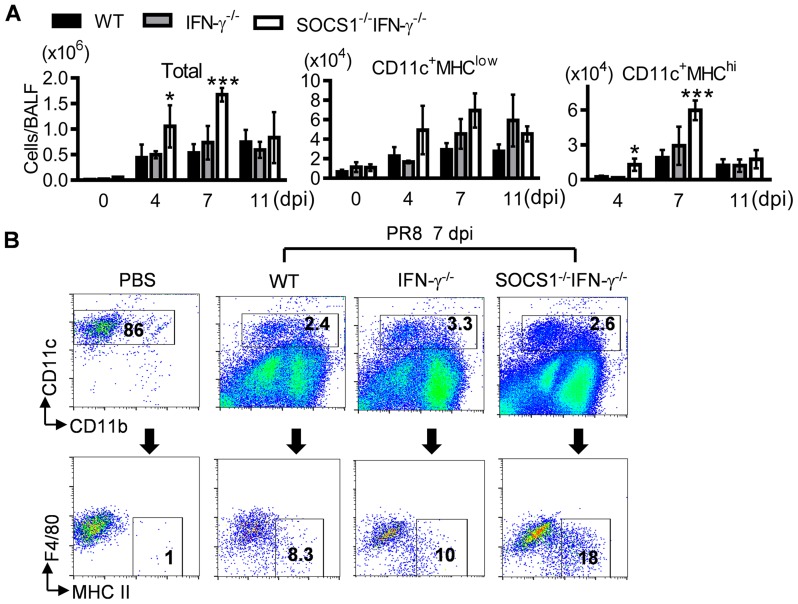
Influenza infection enhances DC recruitment in SOCS1^−/−^IFN-γ^−/−^ mice. (**A**) Numbers of BALF cells (4 mice/group) and (**B**) flow cytometry analysis of airway CD11c^+^ cell subsets at 7 dpi in C57BL/6 WT, IFN-γ^−/−^ and SOCS1^−/−^IFN-γ^−/−^ mice after 50 PFU PR8 infection (3–4 mice/group). In (A) *P*<0.001, ANOVA; *P<0.05, ***, *P*<0.001 relative to WT and IFN-γ^−/−^ mice, Tukey's multiple comparisons test, the data for each time point were repeated in at least two independent experiments. Data in (B) are representative of two independent experiments.

**Figure 3:**
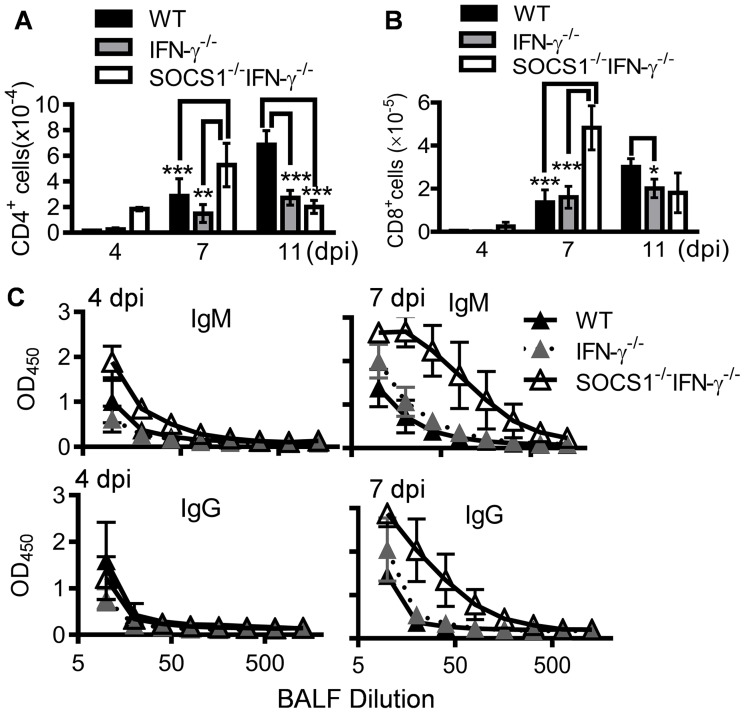
Influenza infection improves adaptive immune responses in SOCS1^−/−^IFN-γ^−/−^ mice. (**A**) Numbers of airway CD4^+^ T cells, (**B**) CD8^+^ T cells, and (**C**) BALF H1N1-specific IgM and IgG levels in C57BL/6 WT, IFN-γ^−/−^ and SOCS1^−/−^IFN-γ^−/−^ mice after 50 PFU PR8 infection (4 mice/group). In (A&B), *P*<0.001, ANOVA; *P<0.05, **, *P*<0.01, ***, *P*<0.001, Tukey's multiple comparisons test. The data for each time point were repeated in at least two independent experiments.

We next sought to determine how SOCS1 may inhibit viral clearance by investigating protective antiviral immune responses in SOCS1^−/−^IFN-γ^−/−^ mice. Alveolar macrophages (CD11c^+^MHC^−^F4/80^+^) are the resident immune population in normal airways (Fig. 2A&B). In response to infection, DCs are recruited to initiate adaptive immunity which is essential for elimination of influenza virus [1], [20]. Consistent with their improved viral clearance, we detected increased numbers of DCs (CD11c^+^MHC^hi^F4/80^low^) in influenza-infected SOCS1^−/−^IFN-γ^−/−^ airways at 7 dpi (Fig. 2A&B). Despite a lack of effect of IFN-γ on viral clearance, IFN-γ^−/−^ mice showed reduced airway T cell numbers compared with WT animals at 11 dpi (Fig. 3A&B), which is in agreement with the regulatory effect of IFN-γ on T cells themselves during influenza infection [21]. Importantly, SOCS1^−/−^IFN-γ^−/−^ mice exhibited significantly increased levels of airway CD4^+^ (Fig. 3A) and CD8^+^ T cells (Fig. 3B), as well as influenza-specific IgM and IgG production at 7 dpi (Fig. 3C). In contrast, peak recruitment of T cells in WT mice was delayed to 11 dpi. Therefore, SOCS1-inhibited viral clearance is associated with delayed adaptive immune responses in IFN-γ^−/−^ and WT mice.

### SOCS1 deficiency results in improved resolution of acute inflammation

**Figure 4:**
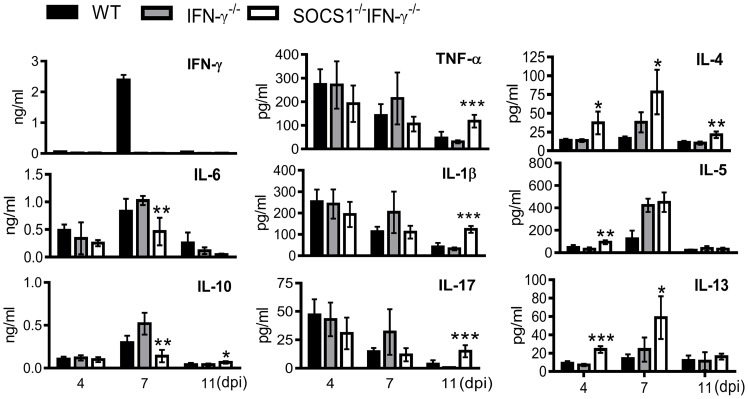
Airway cytokine responses after influenza infection. Levels of IFN-γ, IL-6, IL-10, TNF-α, IL-1β, IL-17, IL-4, IL-5 and IL-13 in BALF of C57BL/6 WT, IFN-γ^−/−^ and SOCS1^−/−^IFN-γ^−/−^ mice after 50 PFU PR8 infection (4 mice/group). *P*<0.001, ANOVA; *P<0.05, **, *P*<0.01, ***, *P*<0.001 relative to WT and IFN-γ^−/−^ mice, Tukey's multiple comparisons test. The data for each time point were repeated in at least two independent experiments.

**Figure 5:**
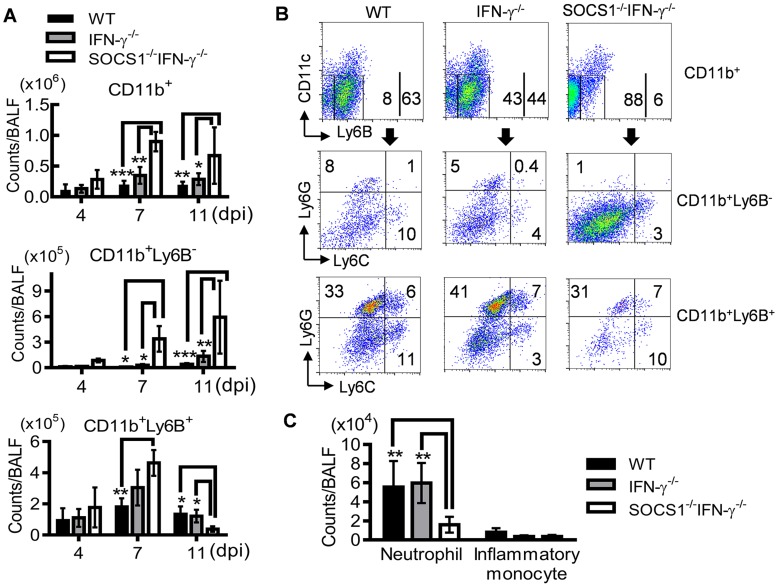
Decreased accumulation of inflammatory cells in SOCS1^−/−^IFN-γ^−/−^ mice at the resolution phase of influenza infection. (**A**) Numbers of CD11b^+^, CD11b^+^Ly6B^−^ and CD11b^+^Ly6B^+^ myeloid cell subsets, (**B**) flow cytometry analysis of airway CD11b^+^, CD11b^+^Ly6B^−^ and CD11b^+^Ly6B^+^ myeloid cell subsets, and (**C**) numbers of CD11b^+^Ly6B^+^Ly6G^+^ neutrophils and CD11b^+^Ly6B^+^Ly6G^−^Ly6C^+^ inflammatory monocytes at 11 dpi in C57BL/6 WT, IFN-γ^−/−^ and SOCS1^−/−^IFN-γ^−/−^ mice after 50 PFU PR8 infection (4 mice/group). In (A), *P*<0.001, ANOVA; *P*<0.05, and **, *P*<0.01, and ***, *P*<0.001, Tukey's multiple comparisons test, the data for each time point were repeated in at least two independent experiments. Data in (B&C) are representative of at least two experiments.

To determine how SOCS1 may exacerbate lung injury, we investigated its regulatory effect on influenza-induced inflammatory responses. SOCS1^−/−^IFN-γ^−/−^ mice exhibited significantly decreased production of IL-6 and IL-10 at 7 dpi but increased IL-4, IL-5 and IL-13 levels in airways during influenza infection (Fig. 4). Surprisingly, TNF-α, IL-1β and IL-17 levels were significantly increased in SOCS1^−/−^IFN-γ^−/−^ mice as compared to IFN-γ^−/−^ and WT animals at 11 dpi (S2 Figure), indicating a disassociation between pro-inflammatory cytokine production and SOCS1-enhanced lung injury at this time point. We next analyzed influenza-induced inflammatory cell infiltration by flow cytometry. Inflammatory cells, including neutrophils and inflammatory monocytes, are recruited into the alveolar space following influenza infection [22]–[24]. Although it remains debatable whether these myeloid cells are essential for immune protection [25], their prolonged presence exacerbates lung damage [26]–[28]. In WT and gene-deficient mice, inflammatory cell infiltrates increased at 7 dpi (Fig. 5A). Interestingly, BALF cell analysis revealed a decrease in the accumulation of CD11b^+^Ly6B^+^ but an increase in CD11b^+^Ly6B^−^ myeloid cells in SOCS1^−/−^IFN-γ^−/−^ mice at 11 dpi (Fig. 5A&B). Since both CD11b^+^Ly6G^+^ neutrophils [29] and CD11b^+^Ly6C^+^ inflammatory monocytes/macrophages [30] comprise the Ly6B-positive subpopulation (Fig. 5B), the reduction in CD11b^+^Ly6B^+^ cells indicates that airway inflammatory cell infiltrates are decreased in SOCS1^−/−^IFN-γ^−/−^ mice. Indeed, SOCS1^−/−^IFN-γ^−/−^ mice exhibited significantly decreased neutrophil numbers at 11 dpi (Fig. 5C). Therefore, SOCS1-enhanced lung injury is consistent with prolonged recruitment of neutrophils in IFN-γ^−/−^ and WT mice during the resolution phase of infection.

### CD8^+^ T cells are sufficient for enhanced viral clearance in SOCS1^−/−^IFN-γ^−/−^ mice

**Figure 6:**
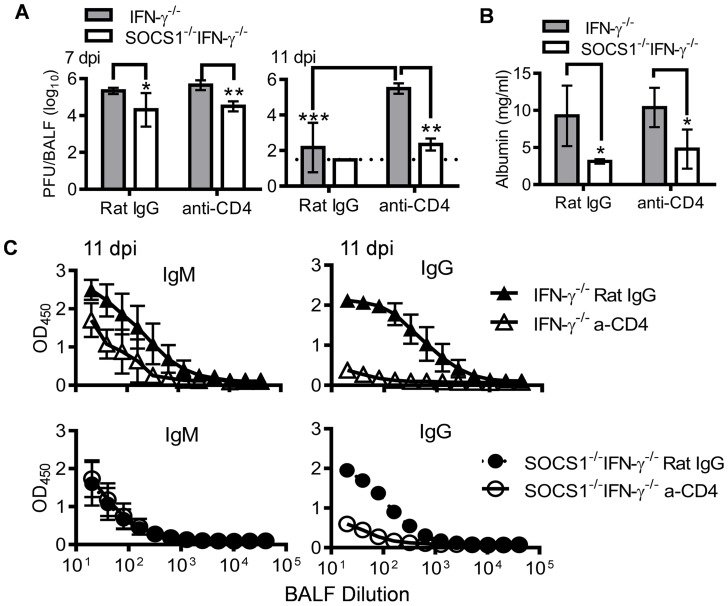
Antiviral immune responses in CD4^+^ T cell depleted mice. (**A**) Viral titers (4–6 mice/group), (**B**) albumin levels (4 mice/group), and (**C**) H1N1-specific IgM and IgG levels (4–6 mice/group) in C57BL/6 IFN-γ^−/−^ and SOCS1^−/−^IFN-γ^−/−^ airways after i.n. infection of 50 PFU PR8 influenza virus. Mice were injected i.p. with GK1.5 (anti-CD4) to deplete CD4^+^ T cells. Control mice were treated with rat IgG. In (A), *P*<0.001, ANOVA; *P<0.05, **, *P*<0.01, ***, *P*<0.001, Tukey's multiple comparisons test, the data for each time point were repeated in at least two independent experiments. In (B), *P<0.05, *t* test. Data in (B&C) are representative of two independent experiments.

It is well established that CD4^+^ T cells play an important role in the elimination of influenza virus, mainly through promoting virus-specific antibody production [31]. We sought to determine the contribution of CD4^+^ T cells to enhanced antiviral immunity in SOCS1^−/−^IFN-γ^−/−^ mice. Anti-CD4 antibody treatment delayed viral clearance in IFN-γ^−/−^ mice but had no significant effect in SOCS1^−/−^IFN-γ^−/−^ animals (Fig. 6A). Conversely, anti-CD4 antibody treatment did not affect the severity of influenza-induced lung injury in either IFN-γ^−/−^ or SOCS1^−/−^IFN-γ^−/−^ mice (Fig. 6B). Importantly, SOCS1^−/−^IFN-γ^−/−^ mice demonstrated significantly improved antiviral immunity compared with IFN-γ^−/−^ mice (Fig. 6A&B), independent of CD4^+^ T cells and influenza-specific IgG production (Fig. 6C). Therefore, although SOCS1^−/−^IFN-γ^−/−^ mice exhibited increased CD4^+^ T cell and influenza-specific antibody responses, these animals were competent in CD4^+^ T cell-independent resolution of influenza infection.

**Figure 7:**
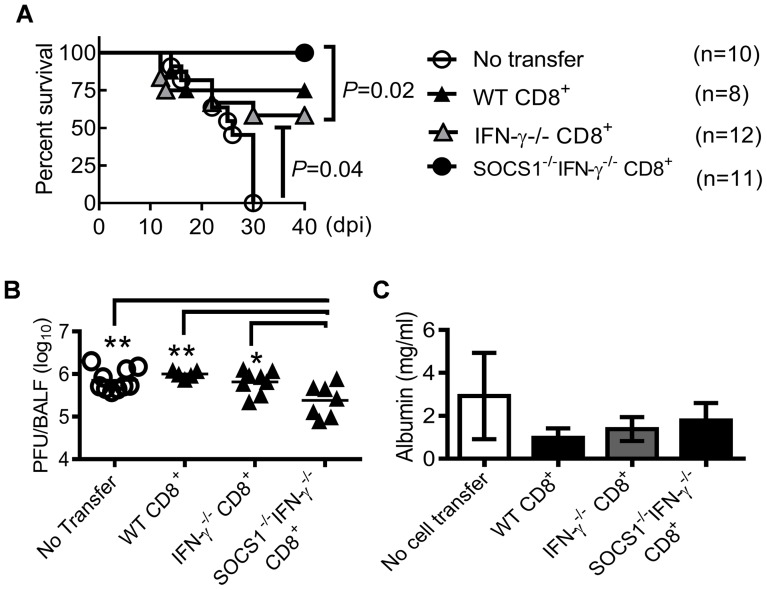
CD8^+^ T cells from SOCS1^−/−^IFN-γ^−/−^ mice protect against influenza infection. (**A**) Survival, (**B**) viral burdens, and (**C**) albumin levels (5–7 mice/group) at 11 dpi in C57BL/6 RAG1^−/−^ mice after i.n. infection of 50 PFU PR8 influenza virus. Mice were i.p injected with 10^7^ CD8^+^ T cells isolated from WT, IFN-γ^−/−^ or SOCS1^−/−^IFN-γ^−/−^ mice 10 days before infection. In (B&C), *P*<0.01, ANOVA; *P<0.05, **, *P*<0.01, Tukey's multiple comparisons test. Data in (A&B) were combined from two independent experiments. Data in (C) are representative of two experiments.

We next investigated whether CD8^+^ T cells in SOCS1^−/−^IFN-γ^−/−^ mice were the effector cells responsible for enhanced viral clearance. Anti-CD8 antibody-treatment depleted CD8^+^ T cells in IFN-γ^−/−^ mice; however, even with an increased dosage, it failed to adequately deplete CD8^+^ T cells in influenza-infected SOCS1^−/−^IFN-γ^−/−^ mice (S3 Figure). Therefore, as an alternative approach, we demonstrated that adoptive transfer of CD8^+^ T cells isolated from naïve SOCS1^−/−^IFN-γ^−/−^ mice rescued RAG1^−/−^ animals from lethal influenza infection (Fig. 7A). This observation was consistent with significantly reduced viral burdens in these animals compared with unreconstituted RAG1^−/−^ controls or RAG1^−/−^ mice reconstituted with WT or IFN-γ^−/−^ T cells (Fig. 7B). Of note, there were limited numbers of virus-specific CD8^+^ T cells in naïve mouse spleens (S4 Figure). We thus conclude that CD8^+^ T cells in SOCS1^−/−^IFN-γ^−/−^ mice are sufficient for improved viral clearance.

### A discrete contribution of SOCS1 to influenza-induced lung injury

**Figure 8:**
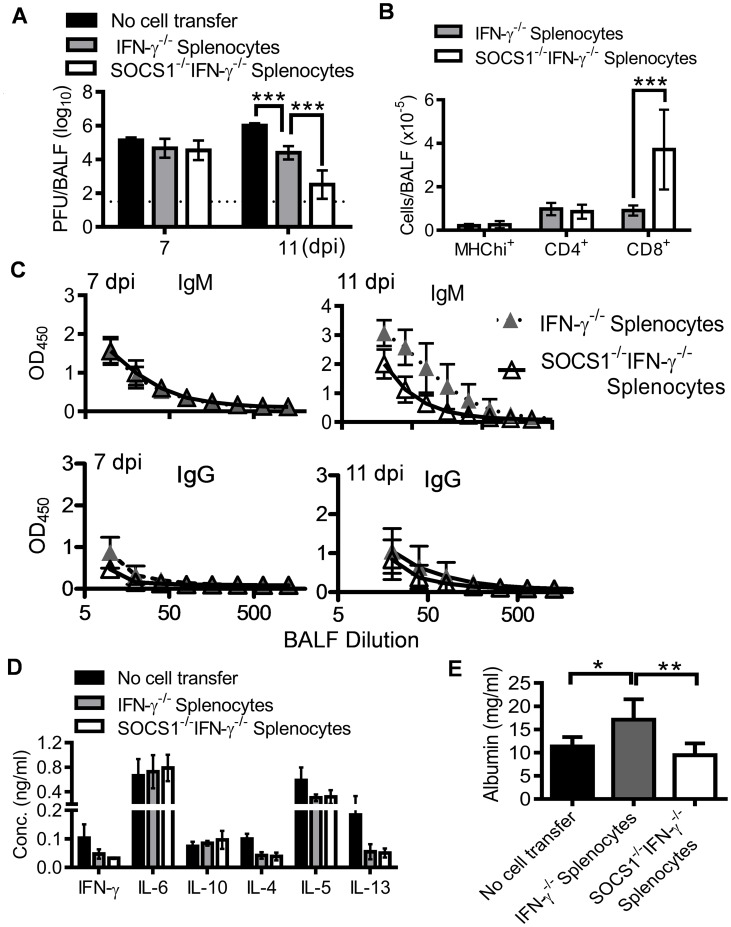
Antiviral immune responses in RAG1^−/−^ mice reconstituted with SOCS1^−/−^IFN-γ^−/−^ adaptive immune cells. (**A**) Airway viral burdens (5–6 mice/group), (**B**) T cell numbers at 7 dpi (4–5 mice/group), (**C**) H1N1-specific antibody responses (4–6 mice/group), (**D**) cytokine responses (5–6 mice/group) at 7 dpi, and (**E**) albumin levels (5–6 mice/group) at 11 dpi in C57BL/6 RAG1^−/−^ mice after i.n. infection of 50 PFU PR8 influenza virus. Mice were i.p injected with 2×10^7^ IFN-γ^−/−^ or SOCS1^−/−^IFN-γ^−/−^ splenocytes 10 weeks before influenza infection. In (A), *P*<0.001, ANOVA; ***, *P*<0.001, Tukey's multiple comparisons test. In (B), ***, *P*<0.001, *t* test. In (C), *P*<0.01, ANOVA; *P<0.05, **, *P*<0.01, Tukey's multiple comparisons test. The data for each time point were repeated in at least two independent experiments.

From the evidence presented above, it appeared that the immunopathological effects of SOCS1 might originate from inadequate viral clearance. However, adoptive transfer of CD8^+^ cells from SOCS1^−/−^IFN-γ^−/−^ mice reduced viral burdens (Fig. 7B) but failed to alleviate lung injury in RAG1^−/−^ mice (Fig. 7C). To determine whether SOCS1 deficiency in other adaptive immune cells is required for optimal viral clearance and thereby amelioration of lung injury, we assessed antiviral immune responses and the severity of influenza-induced lung injury in RAG1^−/−^ mice reconstituted with SOCS1^−/−^IFN-γ^−/−^ adaptive immune cells. SOCS1^−/−^IFN-γ^−/−^ splenocyte recipients demonstrated significantly reduced viral burdens at 11 dpi compared to IFN-γ^−/−^ splenocyte recipients (Fig. 8A), which coincided with increased numbers of CD8^+^ T cells in the airways of the former (Fig. 8B). Conversely, CD4^+^ T cell recruitment (Fig. 8B), antibody production (Fig. 8C), and cytokine responses (Fig. 8D) did not differ significantly between SOCS1^−/−^IFN-γ^−/−^ splenocyte recipients and their corresponding IFN-γ^−/−^ splenocyte recipients. These results indicate that SOCS1 deficiency in adaptive immune cells alone is not sufficient for enhanced CD4^+^ T cell and influenza-specific IgG responses. Importantly, the attenuated lung injury observed in SOCS1^−/−^IFN-γ^−/−^ mice could not be recapitulated by adoptive transfer of their splenocytes (Fig. 8E). This result indicates that SOCS1 expression in innate/stromal cells is involved in enhanced lung injury. Collectively, these data establish a discrete contribution of SOCS1 to lung injury in addition to its inhibitory effect on viral clearance.

## Discussion

Here we demonstrate that mice with a targeted mutation in both SOCS1 and IFN-γ displayed increased adaptive immune responses at the early stage and reduced inflammatory cell infiltration at the resolution stage of influenza infection. These traits led to decreased viral loads and lung injury, and accordingly increased survival rates of SOCS1^−/−^IFN-γ^−/−^ mice following influenza infection. Therefore, in the absence of IFN-γ the cytoplasmic protein SOCS1 not only inhibits viral clearance but also exacerbates inflammatory lung damage. The identification of these non-competing detrimental roles of SOCS1 could have important therapeutic implications for treating influenza infection.

The published evidence demonstrates a role of IFN-γ in regulation of virus-specific CD8^+^ T cell homeostasis following a high dose of (6.8 log10 egg ID50) of A/HKx31 (H3N2) influenza infection [21]. Likewise, we show that IFN-γ^−/−^ mice had reduced airway T cell numbers compared with WT animals at the resolution phase of infection (11 dpi) (Fig. 3A&B). However, we did not detect a significant impact of IFN-γ or SOCS1 on virus-specific CD8^+^ T cell numbers in spleens following 50 PFU of PR8 infection (S5A Figure). Moreover, the percentages of airway CD8^+^ T cells binding to D^b^NP_366_ or D^b^PA_224_ tetramers were comparable among WT, IFN-γ^−/−^ and SOCS1^−/−^IFN-γ^−/−^ mice at 7 dpi (S5B Figure). Of note, these frequencies were significantly increased in all influenza-infected mice at 11 dpi, particularly D^b^NP_366_-specific T cells in SOCS1^−/−^IFN-γ^−/−^ animals (S5B Figure). Interestingly, when only CD8^+^ T cells were available for adaptive antiviral immunity (Fig. 7& S6A Figure), in addition to a small but insignificant (*P* = 0.18) increase in total T cell numbers (S6B Figure), airway virus-specific T cells were significantly increased in RAG1^−/−^ mice that had received SOCS1^−/−^IFN-γ^−/−^ T cells (S6C Figure) as compared to RAG1^−/−^ mice that had received IFN-γ^−/−^ T cells. Together, these results suggest a greater antiviral potential of CD8^+^ T cells from SOCS1^−/−^IFN-γ^−/−^ mice. Given that SOCS1 is an intracellular protein, these immune suppressive effects of SOCS1 are likely T cell-intrinsic. However, we cannot exclude the possibility that SOCS1 expression in other cell types inhibits the effector and expansion potential of naïve CD8^+^ T cells during T cell development.

In addition to enhanced T cell responses, early virus-specific antibody production was also significantly increased in SOCS1^−/−^IFN-γ^−/−^ mice (Fig. 3C). Although in both IFN-γ^−/−^ and SOCS1^−/−^IFN-γ^−/−^ mice, virus-specific IgG production was dependent upon CD4^+^ T cells (Fig. 6C), we detected temporarily increased virus-specific IgM production in SOCS1^−/−^IFN-γ^−/−^ airways independent of CD4^+^ T cells (S7 Figure). This observation is consistent with a previous report showing that early B cell responses to influenza virus is a T cell independent B-1 cell IgM responses [32]. Therefore, we propose that B-1 cell activity is increased in influenza–infected SOCS1^−/−^IFN-γ^−/−^ mice, possibly due to enhanced type I IFN signaling [33]. Likely due to low percentages of B-1 cells in spleens, virus-specific antibody production did not differ significantly between RAG1^−/−^ mice that had received SOCS1^−/−^IFN-γ^−/−^ splenocytes and the corresponding IFN-γ^−/−^ splenocyte recipients (Fig. 8C). Importantly, these data suggest that B-2 cells from SOCS1^−/−^IFN-γ^−/−^ mice are not sufficient for enhanced viral clearance.

In addition to its critical feedback inhibition of IFN-γ signaling, it is noteworthy that SOCS1 is involved in negatively regulating the type I IFN and IFN-lambda signaling pathway [7], [18]. We have previously shown that type I IFN production is induced at the early phase of influenza infection [13]. These findings raise the possibility that the reduced viral burden observed in SOCS1^−/−^IFN-γ^−/−^ mice was due to enhanced IFN antiviral function. In addition, it has also been reported that SOCS1 regulation of type I IFN is responsible for resistance to Semliki Forest virus infection independent of IFN-γ [18]. Moreover, SOCS1-knockdown transgenic mice showed less viral load compared with BALB/c WT mice on day 3 after A/WSN/33 influenza virus infection [7]. However, in the current study, viral burdens at the early phase of infection (4 dpi) were similar among WT, IFN-γ^−/−^ and SOCS1^−/−^IFN-γ^−/−^ mice, suggesting an insignificant effect of SOCS1 on direct innate control of viral replication in epithelial cells. Probably due to the antagonistic effect of influenza virus NS1 protein on type I IFN activity and the redundant role of IFN-lambda in viral clearance [7], [34], BALB/c IFNAR1^−/−^ mice have been shown to be as resistant to influenza infection as WT controls [35]. Conversely, increased susceptibility of C57BL/6 IFNAR1^−/−^ mice to influenza infection results from severe lung inflammation rather than defective viral clearance [24]. Furthermore, using an adoptive transfer system, we showed that CD8^+^ T cells in SOCS1^−/−^IFN-γ^−/−^ mice were sufficient to reduce viral burdens, which is consistent with the reported negative role of SOCS1 during T cell priming and effector functions [36]. Therefore, although a direct antiviral function of type I IFN and IFN-lambda has been demonstrated in other infectious disease models [7], [18], [37], we propose that SOCS1 inhibition of antiviral activities of these IFNs in stromal and innate immune cells is not essential for enhanced viral clearance in SOCS1^−/−^ IFN-γ^−/−^ mice.

Considerable information is now available regarding immune mechanisms essential for elimination of influenza virus [16], [38]. Conversely, little is known about the resolution of inflammation after viral clearance, despite the fact that lung immunopathology is often the main cause of influenza-related morbidity and mortality [17], [39]. Studies in humans and mice suggested that immunological factors critical for efficient viral clearance are also involved in mediating tissue damage [17]. Alternatively, severe immunopathology could be secondary and the consequence of inability to resolve lethal infection [40]. We did not observe a significant correlation between viral loads and lung vascular injury in our non-lethal influenza infection model. Importantly, we found that the detrimental effects of SOCS1 on both viral clearance and lung injury are conveyed through distinct and non-competing cellular pathways as such that, SOCS1 deficiency in adaptive immune cells was sufficient for enhanced viral clearance, while SOCS1 deficiency in innate/stromal cells was required for resolution of pathological damage. Strong evidence to support this possibility came from observations that amelioration of lung injury occurred in SOCS1^−/−^IFN-γ^−/−^ mice after CD4^+^ T cell depletion (Fig. 6B), but not in RAG1^−/−^ animals reconstituted with SOCS1^−/−^IFN-γ^−/−^ adaptive immune cells (Fig. 8E), despite the fact that viral burdens were barely detectable in both groups (Figs. 6A & 8A). Therefore, reduced virus burden alone does not account for the ameliorated immunopathology in influenza-infected SOCS1^−/−^IFN-γ^−/−^ mice. Of note, RAG1^−/−^ mice reconstituted with IFN-γ^−/−^ adaptive immune cells demonstrated increased lung injury following influenza infection. This is consistent with a previous report where CD8^+^ T cell IFN-γ production ameliorated the severity of influenza-induced inflammation and lung damage in an adoptive cell transfer model [41].

SOCS1 is also a critical feedback inhibitor of the IL-4 signaling pathway responsible for anti-inflammatory action and tissue repair [10]. However, it has been shown that CD4^+^ T cell expression of Th2-type cytokines including IL-4 and IL-5 does not promote recovery from viral infection, but rather increases lung viral burdens [42]. Although the role of IL-4 during influenza infection remains debatable, SOCS1^−/−^IFN-γ^−/−^ mice showed increased IL-4 production following influenza infection. Moreover, a positive correlation was observed with IL-4 levels and the severity of lung injury, in that IL-4 production remained elevated in SOCS1^−/−^IFN-γ^−/−^ mice even after CD4^+^ T cell depletion (S8 Figure) but not in RAG1^−/−^ mice reconstituted with SOCS1^−/−^IFN-γ^−/−^ adaptive immune cells (Fig. 8D). Therefore, it is possible that T helper cell-independent IL-4 production is beneficial to the resolution of pathological changes resulting from influenza infection.

As a major anti-inflammatory cytokine, IL-10 has been shown to be crucial in regulating the magnitude of inflammation during influenza infection [43]. Similar to IL-6, peak IL-10 production was significantly decreased in SOCS1^−/−^IFN-γ^−/−^ airways, which correlated with their reduced viral burdens at 7 dpi. In contrast, at the resolution phase of infection, IL-10 level was slightly increased in SOCS1^−/−^IFN-γ^−/−^ mice relative to the low levels in WT and IFN-γ^−/−^ mice, which may contribute to the attenuation of lung inflammation after viral clearance. Moreover, multiple studies have shown that Type I IFN can suppress immune responses by driving production of anti-inflammatory cytokines [44]. Therefore, although we propose that enhanced viral clearance in SOCS1^−/−^IFN-γ^−/−^ mice is not due to increased IFN/STAT1 activation at the acute phase of infection, these SOCS1-regulated pathways may contribute to the resolution of inflammation after viral clearance. We show that SOCS1 in innate/stromal cells is involved in aggravated lung injury, consistent with influenza-induced SOCS1 expression in both cell types (S9 Figure). However, both innate and stromal cells are known to produce as well as respond to anti-inflammatory cytokines. Furthermore, both cell types are directly involved in acute lung injury. Therefore, it would be intriguing albeit challenging, to determine the cellular/molecular pathways responsible for amelioration of lung damage after influenza infection.

The immune suppressive role of SOCS1 had been previously demonstrated in SOCS^+/−^ mice [40], [45]. However, we found these SOCS1-haplodeficient mice were susceptible to influenza infection similar to WT animals (S10 Figure). Although IFN-γ is dispensable for protection against influenza infection, SOCS1-deficient CD8^+^ T cells from SOCS1^−/−^ IFN-γ^−/−^ mice were sufficient to improve antiviral immunity and, furthermore, SOCS1-enhanced lung injury occurred after IFN-γ production was diminished in WT mice, we cannot exclude the possibility that these detrimental effects of SOCS1 were significant only in the absence of T cell IFN-γ production. Nonetheless, to our knowledge, there is no report showing the regulatory role of SOCS1 during the course of influenza infection, and importantly, no evidence directly linking SOCS1 with excessive inflammation in other infectious disease models. The distinct and non-competing detrimental roles of SOCS1, as revealed in this study, make it an appealing target in the design of effective immunotherapies for combating influenza infection.

## Materials and Methods

### Ethics statement

All animal experiments were approved by Albany Medical College Animal Care and Use Committee (Protocol number 11-04004) and University of Nebraska Medical Center (Protocol number 13-057-09-FC), and all experiments were carried out in accordance with Albany Medical College and University of Nebraska Medical Center Assurance of Compliance with PHS Policy on human Care and Use of Laboratory Animals, which is on file with the Office of Protection from Research Risks, NIH.

### Murine model of viral infection

Specific pathogen-free, 8–10 week old C57BL/6 WT, RAG1^−/−^ and IFN-γ^−/−^ mice were purchased from the Jackson Laboratory (Bar Harbor, ME) and bred locally under pathogen-free conditions. C57BL/6 SOCS1^+/−^ mice were obtained from Dr. Warren Alexander (Walter and Eliza Hall Institute) [2], [46] and then mated with C57BL/6 IFN-γ^−/−^ mice from the Jackson Laboratory. Mice were genotyped for deficiency in both SOCS1 and IFN-γ alleles by PCR analysis of genomic DNA isolated from tail tips, using the SOCS3 gene as a positive control (S1A Figure). Primers used for SOCS1: gcatccctcttaacccggtac and aaatgaagccagagaccctcc; for IFN-γ: agaagtaagtggaagggcccagaag and agggaaactgggagaggagaaata; and for SOCS3: gagttttctctgggcgtcctcctag and tggtactcgcttttggagctgaa. Mice deficient in both SOCS1 and IFN-γ alleles were selected and bred locally for the studies. SOCS1^−/−^IFN-γ^−/−^ mice appeared to have normal CD11b^+^ myeloid cell subsets in blood (S1B Figure) and reached to adulthood without any overt signs of pathology [46].

Viral challenge was performed with A/PR8/34 influenza virus (Charles River Laboratories) administered i.n. to anesthetized, sex and age-matched adult mice in 50 µl of sterile PBS. Titers of virus stocks and viral levels in the bronchoalveolar lavage fluids (BALF) and lungs of infected mice were determined by plaque assays on MDCK cell monolayers as previously described [47].

### Flow cytometric analysis

BALF samples were collected by making an incision in the trachea and lavaging the lung twice with 0.8 ml PBS, pH 7.4. Total leukocyte counts were determined using a hemacytometer.

For flow cytometric analysis, BALF cells were incubated with 2.4G2 mAb against FcγRII/III, and stained with PE-Cy7-conjugated anti-CD11c (BD Biosciences), APC-Cy7-conjugated anti-CD11b (BD Biosciences), PE-conjugated Ly6B (Abcom), PCP-Cy5-conjugated anti-Ly6C (eBiosciences) and PE-Cy7 conjugated anti-Ly6G mAb (BD Biosciences) for myeloid cell analysis. APC-conjugated anti-CD11c, APC-Cy7-conjugated anti-CD11b (BD Biosciences), PE-conjugated anti-MHC II (BD Biosciences) and PCP-Cy5-conjugated anti-F4/80 (eBiosciences) were used for DC analysis. PCP-Cy5-conjugated anti-CD3 (Biolegend), APC-conjugated anti-CD4 (BD Biosciences) and APC-Cy7-conjugated anti-CD8 mAb (BD Biosciences) were used for T cell analysis. Splenocytes and BALF cells were stained with Tetramer Alexa 647-Labeled H-2D(b)/PA_224_(SSLENFRAYV) and BV421-Labeled H-2D(b)/NP_366_ (ASNENMETM), using FITC-conjugated anti-CD3 (BD Biosciences), APC-Cy7-conjugated anti-CD4 (BD Biosciences) and PE-conjugated anti-CD8 mAb (BD Biosciences) for cell surface markers. The stained cells were analyzed on a BD FACSCanto or BD LSRII-green using FlowJo and BD FACSDiva software analysis.

### Determination of cytokine production by ELISA

BALF samples were harvested and assayed for TNF-α, IL-1β, IL-6, IL-17, IL-10, IL-4, IL-5, IL-13 and IFN-γ by ELISA using commercially available kits from BD Biosciences and eBioscience (San Diego, CA).

### Determination of viral specific antibody by ELISA

Concentrations of virus-specific antibodies in BALF samples were measured by ELISA. Briefly, Maxisorp ELISA plates (Nalge Nunc International) were coated overnight at 4°C in PBS containing 2 µg/ml Fluvirin (Chiron Vaccines, Liverpool, UK). After washing, 2-fold dilutions of BALF samples were incubated in the plates at 37°C for 2 hr. Detection was performed using biotin-labeled goat anti-mouse IgM or IgG antibodies (Southern Biotechnology Associates, Birmingham, AL). Finally, an avidin-HRP complex (BD Biosciences) was added and OptEIA substrate solution (BD Biosciences) was used for signal development.

### Determination of albumin levels by ELISA

BALF samples were harvested and assayed for albumin by ELISA using a commercially available kit from Bethyl Laboratories (Montgomery, TX).

### T cell depletion

Anti-CD4 and anti-CD8 mAbs were purified from culture supernatants of the GK1.5 and 53-6-72 hybridomas, respectively [13]. C57BL/6 mice were injected i.p. with 500 µg of mAb daily for three days before influenza infection, followed by every three days after infection (18). The efficiency of pulmonary CD4^+^ and CD8^+^ T cell depletion after influenza infection was determined by flow cytometric analysis of isolated BALF cells and splenocytes [13].

### Adoptive cell transfer to RAG1^−/−^

C57BL/6 RAG1^−/−^ mice were injected i.p. with splenocytes (2×10^7^ cells/mouse) isolated from naïve IFN-γ^−/−^ or SOCS1^−/−^IFN-γ^−/−^ mice and infected with 50 PFU PR8 10 weeks after cell transfer [48].

In a separate experiment, total CD8^+^ cells were isolated from the spleens of naïve IFN-γ^−/−^ or SOCS1^−/−^IFN-γ^−/−^ mice through negative magnetic selection using a Mouse CD8^+^ T Cell Isolation Kit (STEMCELL Technologies). The purity of CD8^+^ T Cells was determined to be >90% by flow cytometry. RAG1^−/−^ mice were injected i.p with 10^7^ CD8^+^ cells and infected with PR8 10 days after cell transfer [49], [50]. Influenza-induced airway recruitment of adoptively transferred CD8^+^ T cells was confirmed by flow cytometric analysis.

### Quantitative reverse transcription (RT)-PCR

Alveolar macrophages and BALF dendritic cells were purified using a Mouse CD11c Positive Selection Kit (STEMCELL Technologies). After preparation of single cell suspensions, lung epithelial cells were enriched by negative selection using a Mouse Epithelial Cell Enrichment Kit (STEMCELL Technologies). Total RNA derived from naïve and post-influenza-virus-infected cells was characterized by using iScript Reverse Transcription and iTaqUniversal SYBR Green Supermix (Bio-Rad) on a Bio-Rad CFXConnet Real-Time system. The primer sequences were as following: Fwd 5′- ACAAGCTGCTACAACCAGGG-3′ and Rev 5′-ACTTCTGGCTGGAGACCTCA-3′ for SOCS1; and Fwd 5′-CATAACCTGGTTCATCATCGC-3′ and Rev 5′- GGAGCGGTAGCACCTCCT-3′ for HPRT.

### Statistical analyses

Results are expressed as the mean ± s.d. Significant differences between experimental groups were determined using a Student *t*-test (to compare two samples), or an ANOVA analysis followed by Tukey's multiple comparisons test (to compare multiple samples) in GraphPad Prism 6 (La Jolla, CA). Survival analyses were performed using the Kaplan-Meier log rank test. For all analyses, a *P* value<0.05 was considered to be significant.

## Supporting Information

S1 Figure **Generation of SOCS1^−/−^IFN-γ^−/−^ mice.** (A) Genotyping of gene-deficient mice. Tail DNA was isolated from C57BL/6 WT, IFN-γ^−/−^, and SOCS1^−/−^IFN-γ^−/−^ mice (2 mice/group) and subjected to PCR amplification using primers for SOCS1, IFN-γ and SOCS3 as control. (B) Flow cytometry analysis of CD11b^+^ myeloid cell subsets in blood of 10-week old C57BL/6 WT, IFN-γ^−/−^, and SOCS1^−/−^IFN-γ^−/−^ mice (3 mice/group). The data are representative of at least two experiments.
(DOCX)

S2 Figure **Airway cytokine levels in influenza infected mice.** Levels of IFN-γ, IL-6, IL-10, TNF-α, IL-1β, IL-17, IL-4, IL-5 and IL-13 in BALF of C57BL/6 WT, IFN-γ^−/−^ and SOCS1^−/−^IFN-γ^−/−^ mice after i.n. infection with 50 PFU PR8 influenza virus (4 mice/group). The data for each time point were repeated in at least two independent experiments.
(DOCX)

S3 Figure **Anti-CD8 antibody treatment is inadequate to deplete CD8^+^ T cells in SOCS1^−/−^IFN-γ^−/−^ mice.** The percentages of CD8^+^ T cells in C57BL/6 IFN-γ^−/−^ and SOCS1^−/−^IFN-γ^−/−^ airways on day 7 after i.n. infection with 50 PFU PR8 influenza virus. Mice were injected i.p. with 53-6-72 (anti-CD8) to deplete CD8^+^ T cells. Control mice were treated with rat IgG. Representative plots of cells obtained from 4 different mice in each group. The data are representative of two experiments.
(DOCX)

S4 Figure **Virus-specific CD8^+^ T cells are limited in naïve mouse spleens.** Tetramer staining was used to determine influenza virus-specific CD8^+^ T cells in naïve C57BL/6 WT, IFN-γ^−/−^ and SOCS1^−/−^IFN-γ^−/−^ mice. The percentages of CD8^+^ T cells specific for D^b^NP_366_ or D^b^PA_224_ were determined by flow cytometry. Plots of cells were obtained from 4 pooled spleen samples. Data shown are representative of two independent experiments.
(DOCX)

S5 Figure **Influenza infection induces virus-specific CD8^+^ T cell responses.** Antigen-specific tetramer staining was used to determine CD8^+^ T cell response in mice after i.n. infection with 50 PFU PR8 influenza virus. (**A**) The percentages of CD3^+^CD8^+^ cells specific for D^b^NP_366_ or D^b^PA_224_ in spleens at 11 dpi, and (**B**) in airways at 7 and 11 dpi of C57BL/6 WT, IFN-γ^−/−^ and SOCS1^−/−^IFN-γ^−/−^ mice (4–5 mice/group) was determined by flow cytometry. Splenocytes from naïve SOCS1^−/−^IFN-γ^−/−^ mice were used as a negative control for influenza-induced response. In (B), *P*<0.01, ANOVA; **, *P*<0.01, Tukey's multiple comparisons test. Data shown are representative of two independent experiments.
(DOCX)

S6 Figure **Influenza infection induces CD8^+^ T responses in RAG1^−/−^ mice with adoptive T cell transfer.** (**A**) The percentages of airway CD3^+^CD8^+^ cells (mean ± s.d., 4 mice/group) specific for D^b^NP_366_ or D^b^PA_224_, (**B**) numbers of CD3^+^CD8^+^ cells, and (**C**) combined numbers of CD3^+^CD8^+^ cells specific for D^b^NP_366_ or D^b^PA_224_ in RAG1^−/−^ mice on day 11 after i.n. infection with 50 PFU PR8 influenza virus. Mice were i.p injected with CD8^+^ T cells isolated from IFN-γ^−/−^ or SOCS1^−/−^IFN-γ^−/−^ mice 10 days before infection. In (C), *P<0.05, *t* test. Data shown are representative of two independent experiments.
(DOCX)

S7 Figure **Early H1N1-specific IgM levels in CD4^+^ T cell depleted mice.** H1N1-specific IgM levels in C57BL/6 IFN-γ^−/−^ and SOCS1^−/−^IFN-γ^−/−^ airways on day 7 after i.n. infection with 50 PFU PR8 influenza virus. Mice were injected i.p. with GK1.5 (anti-CD4) to deplete CD4^+^ T cells. Control mice were treated with rat IgG. Data shown are representative of two independent experiments.
(DOCX)

S8 Figure **Airway cytokine responses in CD4^+^ T cell depleted mice.** IL-4 and IL-5 levels in C57BL/6 IFN-γ^−/−^, and SOCS1^−/−^IFN-γ^−/−^ airways on day 7 after i.n. infection with 50 PFU PR8 influenza virus. *, *P*<0.05, **, *P*<0.01, ***, *P*<0.001. Mice were injected i.p. with GK1.5 (anti-CD4) to deplete CD4^+^ T cells. Control mice were treated with rat IgG. Data shown are representative of two independent experiments.
(DOCX)

S9 Figure **Influenza infection induces SOCS1 expression.** Quantitative RT-PCR analysis of SOCS1 transcripts in (**A**) CD11c^+^ BALF cells and (**B**) enriched epithelial cells at 0, 4, 7 and 11 days after influenza virus infection. SOCS1 expression was normalized against hypoxanthine-guanine phosphoribosyltransferase (HPRT). Cells were obtained from 4 pooled lung samples at each time point. Data are representative of two independent experiments.
(DOCX)

S10 Figure **SOCS1^+/−^ mice are susceptible to influenza infection as WT mice.** (**A**) Viral titers in C57BL/6 WT, IFN-γ^−/−^, SOCS1^−/−^IFN-γ^−/−^ and SOCS1^+/−^ airways on day 7 after i.n. infection with 50 PFU PR8 influenza virus. **, *P*<0.01 compared to SOCS1^+/−^ mice. (**B**) Survival of C57BL/6 WT and SOCS1^+/−^ after i.n. infection of 10^3^ PFU PR8 virus. Data shown are representative of two independent experiments.
(DOCX)
