# Supplementary material for: Expression of Suppressor of Cytokine Signaling 1 (SOCS1) Impairs Viral Clearance and Exacerbates Lung Injury during Influenza Infection
Source: PLoS Pathog. 2014 Dec 11;10(12):e1004560. doi: 10.1371/journal.ppat.1004560 (PMC4263766; doi:10.1371/journal.ppat.1004560)
Supplement: S1 Figure — Generation of SOCS1−/−IFN-γ−/− mice. (A) Genotyping of gene-deficient mice. Tail DNA was isolated from C57BL/6 WT, IFN-γ−/−, and SOCS1−/−IFN-γ−/− mice (2 mice/group) and subjected to PCR amplification using primers for SOCS1, IFN-γ and SOCS3 as control. (B) Flow cytometry analysis of CD11b+ myeloid cell subsets in blood of 10-week old C57BL/6 WT, IFN-γ−/−, and SOCS1−/−IFN-γ−/− mice (3 mice/group). The data are representative of at least two experiments. (DOCX) [file ppat.1004560.s001.docx]

**Figure S1 Generation of SOCS1^-/-^IFN-γ^-/-^ mice.** (A) Genotyping of gene-deficient mice. Tail DNA was isolated from C57BL/6 WT, IFN-γ^-/-^, and SOCS1^-/-^IFN-γ^-/-^ mice (2 mice/group) and subjected to PCR amplification using primers for SOCS1, IFN-γ and SOCS3 as control. (B) Flow cytometry analysis of CD11b^+^ myeloid cell subsets in blood of 10-week old C57BL/6 WT, IFN-γ^-/-^, and SOCS1^-/-^IFN-γ^-/-^ mice (3 mice/group). The data are representative of at least two experiments.
