# Supplementary material for: Expression of Suppressor of Cytokine Signaling 1 (SOCS1) Impairs Viral Clearance and Exacerbates Lung Injury during Influenza Infection
Source: PLoS Pathog. 2014 Dec 11;10(12):e1004560. doi: 10.1371/journal.ppat.1004560 (PMC4263766; doi:10.1371/journal.ppat.1004560)
Supplement: S2 Figure — Airway cytokine levels in influenza infected mice. Levels of IFN-γ, IL-6, IL-10, TNF-α, IL-1β, IL-17, IL-4, IL-5 and IL-13 in BALF of C57BL/6 WT, IFN-γ−/− and SOCS1−/−IFN-γ−/− mice after i.n. infection with 50 PFU PR8 influenza virus (4 mice/group). The data for each time point were repeated in at least two independent experiments. (DOCX) [file ppat.1004560.s002.docx]

**Figure S2 Airway cytokine levels in influenza infected mice.** Levels of IFN-γ, IL-6, IL-10, TNF-α, IL-1β, IL-17, IL-4, IL-5 and IL-13 in BALF of C57BL/6 WT, IFN-γ^-/-^ and SOCS1^-/-^IFN-γ^-/-^ mice after i.n. infection with 50 PFU PR8 influenza virus (4 mice/group). The data for each time point were repeated in at least two independent experiments.
