# Supplementary material for: Expression of Suppressor of Cytokine Signaling 1 (SOCS1) Impairs Viral Clearance and Exacerbates Lung Injury during Influenza Infection
Source: PLoS Pathog. 2014 Dec 11;10(12):e1004560. doi: 10.1371/journal.ppat.1004560 (PMC4263766; doi:10.1371/journal.ppat.1004560)
Supplement: S3 Figure — Anti-CD8 antibody treatment is inadequate to deplete CD8+ T cells in SOCS1−/−IFN-γ−/− mice. The percentages of CD8+ T cells in C57BL/6 IFN-γ−/− and SOCS1−/−IFN-γ−/− airways on day 7 after i.n. infection with 50 PFU PR8 influenza virus. Mice were injected i.p. with 53-6-72 (anti-CD8) to deplete CD8+ T cells. Control mice were treated with rat IgG. Representative plots of cells obtained from 4 different mice in each group. The data are representative of two experiments. (DOCX) [file ppat.1004560.s003.docx]

**Figure S3 Anti-CD8 antibody treatment is inadequate to deplete CD8^+^ T cells in SOCS1^-/-^IFN-γ^-/-^ mice.** The percentages of CD8^+^ T cells in C57BL/6 IFN-γ^-/-^ and SOCS1^-/-^IFN-γ^-/-^ airways on day 7 after i.n. infection with 50 PFU PR8 influenza virus. Mice were injected i.p. with 53-6-72 (anti-CD8) to deplete CD8^+^ T cells. Control mice were treated with rat IgG. Representative plots of cells obtained from 4 different mice in each group. The data are representative of two independent experiments.
