# Supplementary material for: Expression of Suppressor of Cytokine Signaling 1 (SOCS1) Impairs Viral Clearance and Exacerbates Lung Injury during Influenza Infection
Source: PLoS Pathog. 2014 Dec 11;10(12):e1004560. doi: 10.1371/journal.ppat.1004560 (PMC4263766; doi:10.1371/journal.ppat.1004560)
Supplement: S4 Figure — Virus-specific CD8+ T cells are limited in naïve mouse spleens. Tetramer staining was used to determine influenza virus-specific CD8+ T cells in naïve C57BL/6 WT, IFN-γ−/− and SOCS1−/−IFN-γ−/− mice. The percentages of CD8+ T cells specific for DbNP366 or DbPA224 were determined by flow cytometry. Plots of cells were obtained from 4 pooled spleen samples. Data shown are representative of two independent experiments. (DOCX) [file ppat.1004560.s004.docx]

**Figure S4 Virus-specific CD8^+^ T cells are limited in naïve mouse spleens**. Tetramer staining was used to determine influenza virus-specific CD8^+^ T cells in naïve C57BL/6 WT, IFN-γ^-/-^ and SOCS1^-/-^IFN-γ^-/-^ mice. The percentages of CD8^+^ T cells specific for D^b^NP_366_ or D^b^PA_224_ were determined by flow cytometry. Plots of cells were obtained from 4 pooled spleen samples. Data shown are representative of two independent experiments.
