# Supplementary material for: Expression of Suppressor of Cytokine Signaling 1 (SOCS1) Impairs Viral Clearance and Exacerbates Lung Injury during Influenza Infection
Source: PLoS Pathog. 2014 Dec 11;10(12):e1004560. doi: 10.1371/journal.ppat.1004560 (PMC4263766; doi:10.1371/journal.ppat.1004560)
Supplement: S5 Figure — Influenza infection induces virus-specific CD8+ T cell responses. Antigen-specific tetramer staining was used to determine CD8+ T cell response in mice after i.n. infection with 50 PFU PR8 influenza virus. (A) The percentages of CD3+CD8+ cells specific for DbNP366 or DbPA224 in spleens at 11 dpi, and (B) in airways at 7 and 11 dpi of C57BL/6 WT, IFN-γ−/− and SOCS1−/−IFN-γ−/− mice (4–5 mice/group) was determined by flow cytometry. Splenocytes from naïve SOCS1−/−IFN-γ−/− mice were used as a negative control for influenza-induced response. In (B), P<0.01, ANOVA; **, P<0.01, Tukey's multiple comparisons test. Data shown are representative of two independent experiments. (DOCX) [file ppat.1004560.s005.docx]

**Figure S5 Influenza infection induces virus-specific CD8^+^ T cell responses.** Antigen-specific tetramer staining was used to determine CD8^+^ T cell responses in mice after i.n. infection with 50 PFU PR8 influenza virus. **(A)** The percentages of CD3^+^CD8^+^ cells specific for D^b^NP_366_ or D^b^PA_224_ in spleens at 11 dpi, and **(B)** in airways at 7 and 11 dpi of C57BL/6 WT, IFN-γ^-/-^ and SOCS1^-/-^IFN-γ^-/-^ mice (4-5 mice/group) was determined by flow cytometry. Splenocytes from naïve SOCS1^-/-^IFN-γ^-/-^ mice were used as a negative control for influenza-induced responses. In (B), *P*<0.01, ANOVA; ***, P<* 0.01, Tukey's multiple comparisons test. Data shown are representative of two independent experiments.
