# Supplementary material for: Expression of Suppressor of Cytokine Signaling 1 (SOCS1) Impairs Viral Clearance and Exacerbates Lung Injury during Influenza Infection
Source: PLoS Pathog. 2014 Dec 11;10(12):e1004560. doi: 10.1371/journal.ppat.1004560 (PMC4263766; doi:10.1371/journal.ppat.1004560)
Supplement: S6 Figure — Influenza infection induces CD8+ T responses in RAG1−/− mice with adoptive T cell transfer. (A) The percentages of airway CD3+CD8+ cells (mean ± s.d., 4 mice/group) specific for DbNP366 or DbPA224, (B) numbers of CD3+CD8+ cells, and (C) combined numbers of CD3+CD8+ cells specific for DbNP366 or DbPA224 in RAG1−/− mice on day 11 after i.n. infection with 50 PFU PR8 influenza virus. Mice were i.p injected with CD8+ T cells isolated from IFN-γ−/− or SOCS1−/−IFN-γ−/− mice 10 days before infection. In (C), *P<0.05, t test. Data shown are representative of two independent experiments. (DOCX) [file ppat.1004560.s006.docx]

**Figure S6 Influenza infection induces CD8^+^ T responses in RAG1^-/-^ mice after adoptive T cell transfer. (A)** The percentages of airway CD3^+^CD8^+^ cells (mean + s.d., 4 mice/group) specific for D^b^NP_366_ or D^b^PA_224_, **(B)** numbers of CD3^+^CD8^+^ cells, and **(C)** combined numbers of CD3^+^CD8^+^ cells specific for D^b^NP_366_ or D^b^PA_224_ in reconstituted RAG1^-/-^ mice on day 11 after i.n. infection with 50 PFU PR8 influenza virus. Mice were i.p injected with CD8^+^ T cells isolated from IFN-γ^-/-^ or SOCS1^-/-^IFN-γ^-/-^ mice 10 days before infection. In (C), **P*< 0.05, *t* test. Data shown are representative of two independent experiments.
