# Supplementary material for: Expression of Suppressor of Cytokine Signaling 1 (SOCS1) Impairs Viral Clearance and Exacerbates Lung Injury during Influenza Infection
Source: PLoS Pathog. 2014 Dec 11;10(12):e1004560. doi: 10.1371/journal.ppat.1004560 (PMC4263766; doi:10.1371/journal.ppat.1004560)
Supplement: S8 Figure — Airway cytokine responses in CD4+ T cell depleted mice. IL-4 and IL-5 levels in C57BL/6 IFN-γ−/−, and SOCS1−/−IFN-γ−/− airways on day 7 after i.n. infection with 50 PFU PR8 influenza virus. *, P<0.05, **, P<0.01, ***, P<0.001. Mice were injected i.p. with GK1.5 (anti-CD4) to deplete CD4+ T cells. Control mice were treated with rat IgG. Data shown are representative of two independent experiments. (DOCX) [file ppat.1004560.s008.docx]

**Figure S8 Airway cytokine responses in CD4^+^ T cell depleted mice.** IL-4 and IL-5 levels in C57BL/6 IFN-γ^-/-^, and SOCS1^-/-^IFN-γ^-/-^ airways on day 7 after i.n. infection with 50 PFU PR8 influenza virus (4 mice/group). **, P<* 0.05*,* ***, P<* 0.01, ****, P<*0.001. Mice were injected i.p. with GK1.5 (anti-CD4) to deplete CD4^+^ T cells. Control mice were treated with rat IgG. Data shown are representative of two independent experiments.
