# Supplementary material for: Expression of Suppressor of Cytokine Signaling 1 (SOCS1) Impairs Viral Clearance and Exacerbates Lung Injury during Influenza Infection
Source: PLoS Pathog. 2014 Dec 11;10(12):e1004560. doi: 10.1371/journal.ppat.1004560 (PMC4263766; doi:10.1371/journal.ppat.1004560)
Supplement: S9 Figure — Influenza infection induces SOCS1 expression. Quantitative RT-PCR analysis of SOCS1 transcripts in (A) CD11c+ BALF cells and (B) enriched epithelial cells at 0, 4, 7 and 11 days after influenza virus infection. SOCS1 expression was normalized against hypoxanthine-guanine phosphoribosyltransferase (HPRT). Cells were obtained from 4 pooled lung samples at each time point. Data are representative of two independent experiments. (DOCX) [file ppat.1004560.s009.docx]

**Figure S9 Influenza infection induces SOCS1 expression.** Quantitative RT-PCR analysis of SOCS1 transcripts in **(A)** CD11c^+^ BALF cells and **(B)** enriched epithelial cells at 0, 4, 7 and 11 days after influenza virus infection. SOCS1 expression was normalized against hypoxanthine-guanine phosphoribosyltransferase (HPRT). Cells were obtained from 4 pooled lung samples at each time point. Data are representative of two independent experiments.
