# Supplementary material for: Expression of Suppressor of Cytokine Signaling 1 (SOCS1) Impairs Viral Clearance and Exacerbates Lung Injury during Influenza Infection
Source: PLoS Pathog. 2014 Dec 11;10(12):e1004560. doi: 10.1371/journal.ppat.1004560 (PMC4263766; doi:10.1371/journal.ppat.1004560)
Supplement: S10 Figure — SOCS1+/− mice are susceptible to influenza infection as WT mice. (A) Viral titers in C57BL/6 WT, IFN-γ−/−, SOCS1−/−IFN-γ−/− and SOCS1+/− airways on day 7 after i.n. infection with 50 PFU PR8 influenza virus. **, P<0.01 compared to SOCS1+/− mice. (B) Survival of C57BL/6 WT and SOCS1+/− after i.n. infection of 103 PFU PR8 virus. Data shown are representative of two independent experiments. (DOCX) [file ppat.1004560.s010.docx]

**Figure S10** **SOCS1^+/-^ mice are susceptible to influenza infection as WT mice. (A)** Viral titers in C57BL/6 WT, IFN-γ^-/-^, SOCS1^-/-^IFN-γ^-/-^ and SOCS1^+/-^ airways on day 7 after i.n. infection of 50 PFU PR8 influenza virus. ***, P<* 0.01 compared to SOCS1^+/-^ mice. **(B)** Survival of C57BL/6 WT and SOCS1^+/-^ after i.n. infection of 10^3^ PFU PR8 virus. Data shown are representative of two independent experiments.
